# AMPK – roles in nutrient sensing and in cancer

**DOI:** 10.1186/2049-3002-2-S1-O16

**Published:** 2014-05-28

**Authors:** D Grahame Hardie

**Affiliations:** 1 College of Life Sciences, University of Dundee, Dundee, DD1 5EH, Scotland, UK

## 

The AMP-activated protein kinase (AMPK) is a central sensor of cellular energy status. The kinase is activated by increases in cellular AMP by three mechanisms: (i) allosteric activation; (ii) promotion of Thr172 phosphorylation by the upstream kinase LKB1; (iii) inhibition of Thr172 dephosphorylation. Of these, only the third is mimicked by ADP, but all three are antagonized by ATP, thus allowing the system to act as a sensor of cellular energy.

Genes encoding the three subunits of AMPK are found in essentially all eukaryotes, and in unicellular eukaryotes such as *Saccharomyces cerevisiae* the kinase has a role in sensing and responding to glucose starvation. This cell-autonomous role has been preserved in multicellular eukaryotes, but the system has also adapted to respond to hormones involved in regulating whole body energy balance, such as ghrelin, leptin and adiponectin. This suggests that AMPK would be involved in disorders of energy balance, especially insulin resistance. Indeed, AMPK is activated by metformin, currently the first choice drug for treatment of type 2 diabetes. Metformin activates AMPK indirectly by inhibiting the respiratory chain, and does have AMPK-independent effects. However, recent studies suggest that its long-term insulin-sensitizing effects are mediated by AMPK activation and consequent effects on lipid metabolism that lower intracellular di- and tri-glycerides.

Findings that the tumor suppressor LKB1 was the upstream kinase phosphorylating Thr172, which is required for AMPK activation by energy stress, introduced a link between AMPK and cancer. Given the importance to cancer of metabolic changes such as increased lipid, protein and RNA synthesis, and the switch from oxidative metabolism to glycolysis (all opposed by AMPK) it seems likely that AMPK exerts some of the tumor suppressor effects of LKB1. One would therefore expect a strong selection pressure for mechanisms that down-regulate AMPK activation. Loss-of-function mutations in LKB1 (found in lung, cervical and skin cancer) is one way this can happen, while reduced expression of AMPK-α2 is also observed in hepatocellular carcinomas. We are currently studying the role of the “ST loop”, a serine/threonine-rich region present in the C-terminal domains of all vertebrate AMPK-α subunits. This loop is phosphorylated at multiple sites by growth-associated kinases such as Akt, and this inhibits phosphorylation of Thr172, and hence AMPK activation, by mechanisms that will be discussed. Thus, a previously unrecognized effect of hyper-activation of Akt in tumor cells is that the antagonistic effects of AMPK on cell growth and biosynthesis are reduced.

